# Nanoassembly routes stimulate conflicting antibody quantity and quality for transmission-blocking malaria vaccines

**DOI:** 10.1038/s41598-017-03798-3

**Published:** 2017-06-19

**Authors:** Darren B. Leneghan, Kazutoyo Miura, Iona J. Taylor, Yuanyuan Li, Jing Jin, Karl D. Brune, Martin F. Bachmann, Mark Howarth, Carole A. Long, Sumi Biswas

**Affiliations:** 1 Jenner Institute, University of Oxford, Oxford, OX3 7DQ UK; 2 Laboratory of Malaria and Vector Research, National Institute of Allergy and Infectious Diseases/National Institutes of Health, Rockville, Maryland 20852 USA; 3 Department of Biochemistry, University of Oxford, South Parks Road, Oxford, OX1 3QU UK; 4 Inselspital, University of Bern, Bern, Switzerland

## Abstract

Vaccine development efforts have recently focused on enabling strong immune responses to poorly immunogenic antigens, via display on multimerisation scaffolds or virus like particles (VLPs). Typically such studies demonstrate improved antibody titer comparing monomeric and nano-arrayed antigen. There are many such studies and scaffold technologies, but minimal side-by-side evaluation of platforms for both the amount and efficacy of antibodies induced. Here we present direct comparison of three leading platforms displaying the promising malaria transmission-blocking vaccine (TBV) target Pfs25. These platforms encompass the three important routes to antigen-scaffold linkage: genetic fusion, chemical cross-linking and plug-and-display SpyTag/SpyCatcher conjugation. We demonstrate that chemically-conjugated Qβ VLPs elicited the highest *quantity* of antibodies, while SpyCatcher-AP205-VLPs elicited the highest *quality* anti-Pfs25 antibodies for transmission blocking upon mosquito feeding. These quantative and qualitative features will guide future nanoassembly optimisation, as well as the development of the new generation of malaria vaccines targeting transmission.

## Introduction

The development of effective vaccines is recognised as one of the greatest achievements in medicine^1^. Historically, most vaccines have been attenuated or inactivated formulations of whole-organisms given prophylactically. In the modern vaccination age, the pathway to licensure and deployment can be significantly more difficult for these types of complex whole-organism vaccine as they sometimes induce strong reactogenicity^2^ in vaccinees such as high fever, nausea and headache. This is particularly undesirable in low-income settings where symptoms of reactogenicity can be confused for symptoms of disease, straining already minimal healthcare resources. These factors have spurred a move in the focus of both academic research institutions and the pharmaceutical industry towards defined subunit-based vaccines.

Subunit vaccines typically comprise purified antigens or parts of antigens (sub-domains or peptides) that elicit a protective immune response^3^. The disadvantage of this approach to vaccination however, is that on their own subunit antigens are often not immunogenic enough to elicit a protective immune response^4^. For this reason subunit vaccines generally incorporate some form of immunostimulant. One such immunostimulant that has recently “come of age”^5^ is the use of self-assembling protein nanoparticles, commonly multimerisation domains or virus-like particles (VLPs) to display the antigen of interest. VLPs are made by recombinantly expressing coat, envelope or capsid viral proteins in a heterologous system and are able to self-assemble into particulate structures which take advantage of the key immunologic features of viruses such as repetitive surfaces and activation of pathogen-associated molecular pattern recognition receptors^5^. As well as being able to induce a strong response against the virus from which they are derived, VLPs can also be used to display foreign antigens by either genetic fusion to the coat protein or other means of conjugation. Display of foreign antigens to the immune system in this manner is able to elicit much stronger cellular and humoral immunity than soluble antigen alone^6, 7^.

Transmission blocking malaria vaccines (TBVs) aim to inhibit sexual reproduction of the malaria parasite by eliciting antibodies which prevent the parasite from replicating within the mosquito vector. Antibodies generated by vaccination with the *Plasmodium falciparum* malaria transmission blocking antigen Pfs25 have been widely reported to be able to completely inhibit parasite development^4, 8–11^ making it an exciting TBV candidate. A successful TBV would be an important supplement to traditional malaria control strategies in countries seeking to ease the burden of endemic malaria^12^. As TBVs rely solely on antibodies for their functional action^13^, TBVs are an excellent model to assess differences in functional efficacy elicited by different immunostimulant scaffolds.

Numerous studies have detailed novel VLPs^14–19^ and their immunogenic properties, however it is unclear whether every VLP is equally effective. There are several studies reporting that antigens conjugated to VLPs are significantly more immunogenic than soluble antigen alone^6, 20^ but side-by-side comparison of the same antigen on several VLP platforms has not been reported. Though some studies have compared VLPs to other scaffolds such as genetically detoxified diphtheria toxin^21^, spherical vs. rod shaped VLP assemblies^22^, as well as the adjuvant ability of various polysaccharide carriers^23^.

In this study we have compared three leading platforms displaying Pfs25. These platforms comprise the three important routes to antigen-scaffold linkage: genetic fusion, chemical cross-linking and plug-and-display SpyTag/SpyCatcher. The first, IMX313, is a multimerisation domain based on a hybrid version of the chicken complement inhibitor C4b-binding protein (C4bp). Previous work has shown IMX313 fused to various antigens increases antibody titer and avidity as well as improving antigen localisation to lymph nodes and improving cellular immunogenicity^4, 24^. The second, SpyCatcher-AP205, is a novel VLP strategy we recently established^6, 25^ which allows covalent conjugation of antigen to VLP by the formation of a spontaneous isopeptide bond between SpyCatcher and its partner SpyTag^26^. Fusion of SpyCatcher to the VLP and SpyTag to the antigen allows heterologous expression of antigen and VLP in the system most suited to each and avoids the typical downfalls of antigen-fusion VLPs such as poor expression levels, poor solubility and misfolding. This system has shown promising improvements in antigen immunogenicity compared to monomeric antigen in adjuvant^6^. Finally, chemical crosslinking of antigen to VLPs consisting of the coat protein of the bacteriophage Qβ has been shown to improve immunogenicity in disease areas such as influenza^27^, malaria^28^, and Alzheimer’s^29^, as well as nicotine addiction^30^ and diabetes^31^.

Herein we determine that there are key differences in the improved immunogenicity between the three immunostimulant scaffolds. Further we demonstrate a discordance between the antibody titer induced by each platform and the functional efficacy of those antibodies.

## Results

### Generation and biophysical analysis of nanoparticle vaccines

**Figure 1:**
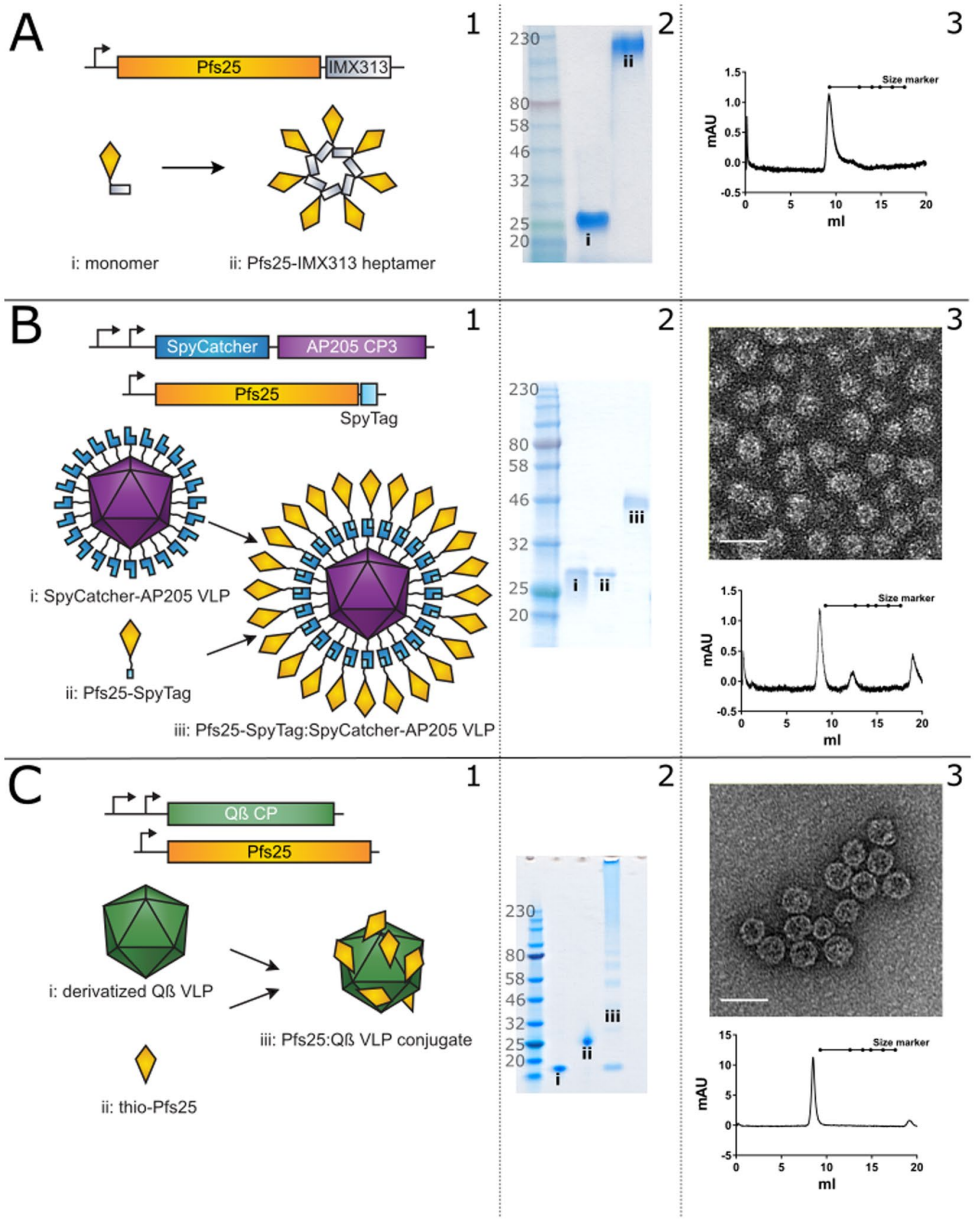
Generation and characterisation of nanoparticles used in this study. (**A**) **Pfs25-IMX313** (1). Schematic of construct design particle and formation illustrating monomer (i) and heptamer (ii). (2) SDS-PAGE showing reduced monomer (i) and non-reduced heptamer (ii). (3) Size exclusion chromatography trace illustrating heptamer migration in the expected range. (**B**) **Pfs25-SpyTag:SpyCatcher-AP205** (1) Schematic of construct design and particle formation illustrating SpyCatcher-AP205 (i), Pfs25-SpyTag (ii), and Pfs25-SpyTag:SpyCatcher-AP205 conjugate (iii). (2) Reducing SDS-PAGE showing SpyCatcher-AP205 (i), Pfs25-SpyTag (ii), and Pfs25-SpyTag:SpyCatcher-AP205 conjugate (iii). (3) Negatively stained TEM of Pfs25-SpyTag:SpyCatcher-AP205, scale bar = 50 nm and size exclusion chromatography trace illustrating VLP migration in the expected range. (**C**) **Pfs25-Qβ** (1) Schematic of construct design and particle formation illustrating Qβ VLPs (i), Pfs25 (ii), and Pfs25-Qβ conjugate (iii). (2) Reducing SDS-PAGE showing Qβ VLPs (i), Pfs25 (ii), and Pfs25-Qβ conjugate (iii). (3) Negatively stained TEM of Pfs25-Qβ VLPs, scale bar = 50 nm and size exclusion chromatography trace illustrating VLP migration in the expected range. Numbers at the left of the coomassie images represent size in kDa. Size markers in all size exclusion traces are from left to right: 670, 158, 75, 44, 29 and 13.7 kDa.

Pfs25-IMX313 and Pfs25-SpyTag:SpyCatcher-AP205 were successfully expressed and purified as previously described (Fig. 1A and B)^4, 6^. Pfs25 coupled to Qβ by chemical crosslinking (Fig. 1C) was analysed by Coomassie-stained reducing (Fig. 1C) and non-reducing (Fig. S1) SDS-PAGE, as well as by Western blot using Pfs25-specific monoclonal antibodies (Fig. S1). Particle integrity was confirmed by size exclusion chromatography (Fig. 1C) and electron microscopy (Fig. 1C). Qβ coat protein migrated at the expected size of ~14 kDa in its reduced form. Addition of maleimide groups (derivatisation) induced a band pattern consistent with internally crosslinked multimers corresponding to 2×, 3×, 4× Qβ (Fig. S1). The non-reduced forms of both pre- and post-derivatisation Qβ are consistent with the formation of higher order oligomers. Addition of sulfhydryl groups to Pfs25 did not appear to significantly change its migration pattern on SDS-PAGE, though recognition of the non-reduced form of thio-Pfs25 was recognised more strongly than the non-reduced form of untreated Pfs25 by both mAbs (4B7 and 32F81) suggesting addition of sulfhydryl groups may partially unfold the structure of the protein (Fig. S1). These mAbs have previously been described to detect the reduced and non-reduced forms of Pfs25 with different intensity^32, 33^. Successful crosslinking of one Pfs25 molecule to one Qβ molecule should result in a monomer of approximately 32 kDa. A band of approximately the correct size (indicated by (iii)) is evident on the Coomassie-stained gel (Fig. 1 C2) as well as the Western blots (Fig. S1), indicating successful conjugation. Higher order conjugates are also evident (Fig. S1).

To determine the conjugation efficiency of Pfs25 to Qβ, the Coomassie-stained gel was analysed by densitometry. By comparing the relative pixel intensity between the Qβ monomer and Qβ-Pfs25 and adjusting for the change in molecular weight, the efficiency of coupling was estimated to be between 7% and 11% for a number of different coupling experiments. Given that each Qβ particle is made up of 180 monomers^34^, this gives between 13 and 20 Pfs25 monomers per particle. This is the minimum conjugation efficiency; evident higher order bands on both Western blot and Coomassie-stained gel mean that the true number of Pfs25 monomers per particle is in reality much higher but due to the indistinct nature of the bands it is impossible to accurately estimate by densitometry. The dose required for immunisation was calculated based on this minimum conjugation efficiency. So as not to unfairly bias towards Pfs25-Qβ, 25% of the calculated dose (3 μg of Pfs25-Qβ) was used; comparison by polyclonal stained Western blot of 1 µg of Pfs25-Qβ against 1 µg of Pfs25 (data not shown) was used as a guide for this estimate.

Electron microscopy of the VLP preparations revealed intact Pfs25-Qβ VLPs and Pfs25-SpyTag:SpyCatcher-AP205 VLPs of approximately the expected size, ~35 nm (Fig. 1, B3 and C3). Size exclusion chromatography of Pfs25-Qβ and Pfs25-SpyTag:SpyCatcher-AP205 demonstrated elution profiles in the expected range for VLPs (Fig. 1, B3 and C3), while the elution profile for Pfs25-IMX313 is consistent with the size previously reported^4^.

### Nanoparticle vaccines are strongly immunogenic

**Figure 2:**
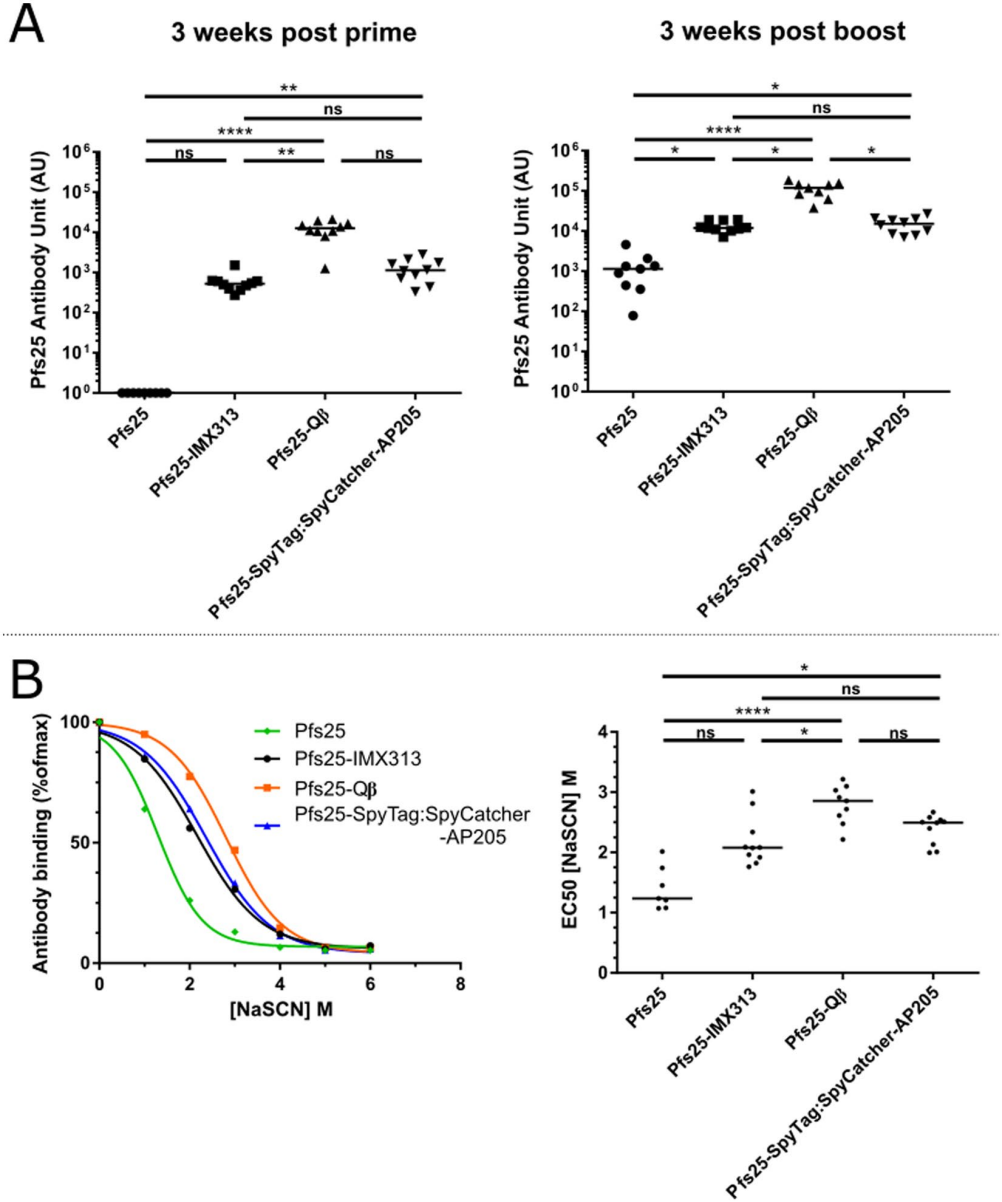
Immunogenicity of nanoparticle vaccines. (**A**) Anti-Pfs25 IgG titers measured by ELISA in mice (n = 10) vaccinated twice with doses corresponding to 1 μg Pfs25 per immunisation in Alhydrogel® at three weeks post prime (left panel) and three weeks post boost (right panel). (**B)** Avidity of serum IgG responses was assessed by NaSCN-displacement ELISA and is reported as the reduction in OD by incubation with varying concentrations of NaSCN compared to incubation with PBS (left panel). Lines represent the arithmetic mean of the combined data from each mouse within the group. EC50 (concentration of NaSCN required to reduce the OD405 to 50% of that without NaSCN) was then determined using a sigmoidal dose response (variable slope) regression (right panel). ns p > 0.05, *p ≤ 0.05, **p ≤ 0.01, ***p ≤ 0.001, ****p ≤ 0.0001.

Groups of 10 mice were immunised with doses normalised to 1 μg Pfs25 antigen content and formulated in Alhydrogel (Table 1). Three weeks after the priming immunisation, mice in each of the groups, except those that had received monomeric Pfs25, showed detectable level of antibody responses (Fig. 2A). Following the boosting dose, each of the groups demonstrated some response to Pfs25. The poorest response unsurprisingly came from the group of mice immunised with monomeric Pfs25 in Alhydrogel.

**Table 1:** Vaccine origin and dose details.

Antigen	Produced in	Dose: Pfs25 µg (total protein µg)
Pfs25	*P. pastoris*	1 (1)
Pfs25-IMX313	*P. pastoris*	1 (1.35)
Pfs25-Qβ	*P. pastoris* and *E. coli*	1 (3)
Pfs25-SpyTag:SpyCatcher-AP205	HEK293E and *E. coli*	1 (2.24)

Of the groups that received nanoparticle vaccines, Pfs25-SpyTag:SpyCatcher-AP205 and Pfs25-Qβ had a significantly higher anti-Pfs25 antibody titer than those that received monomeric Pfs25 after the priming immunisation (Pfs25-SpyTag:SpyCatcher-AP205 p < 0.001, Pfs25- Qβ p < 0.0001). Subsequently all three nanoparticle groups had a significantly higher anti-Pfs25 antibody titer post boost (Pfs25-SpyTag:SpyCatcher-AP205 p < 0.05, Pfs25-Qβ p < 0.0001, Pfs25-IMX313 p < 0.05). Comparing only within the groups that received the nanoparticle vaccines the Pfs25-Qβ group had significantly higher antibody response than Pfs25-IMX313 post prime (p < 0.01) and significantly higher response than both Pfs25-IMX313 (p < 0.05) and Pfs25-SpyTag:SpyCatcher-AP205 (p < 0.05) post boost. There was no significant difference between the Pfs25-IMX313 and Pfs25-SpyTag:SpyCatcher-AP205 groups at any time point measured (p > 0.05, Fig. 2A).

Antibody avidity was measured using the chaotropic agent sodium thiocyanate (NaSCN) to displace serum antibodies in ELISA^35^. Using this method we determined for each the avidity EC50 (amount of NaSCN required to decrease OD in the ELISA by 50%). The mice that received Pfs25-Qβ generated an antibody response with significantly higher avidity than both the monomeric Pfs25 and Pfs25-IMX313 groups. The Pfs25-Qβ immunised mice did not develop significantly higher avidity antibodies than the Pfs25-SpyTag:SpyCatcher-AP205 (p > 0.05) group although there is a trend towards higher avidity. Both the Pfs25-Qβ (p < 0.001) and Pfs25-SpyTag:SpyCatcher-AP205 (p < 0.05) immunised groups generated a significantly higher avidity antibodies than monomeric Pfs25 immunised mice (Fig. 2B).

### Vaccine-induced antibodies recognise native Pfs25 antigen

**Figure 3:**
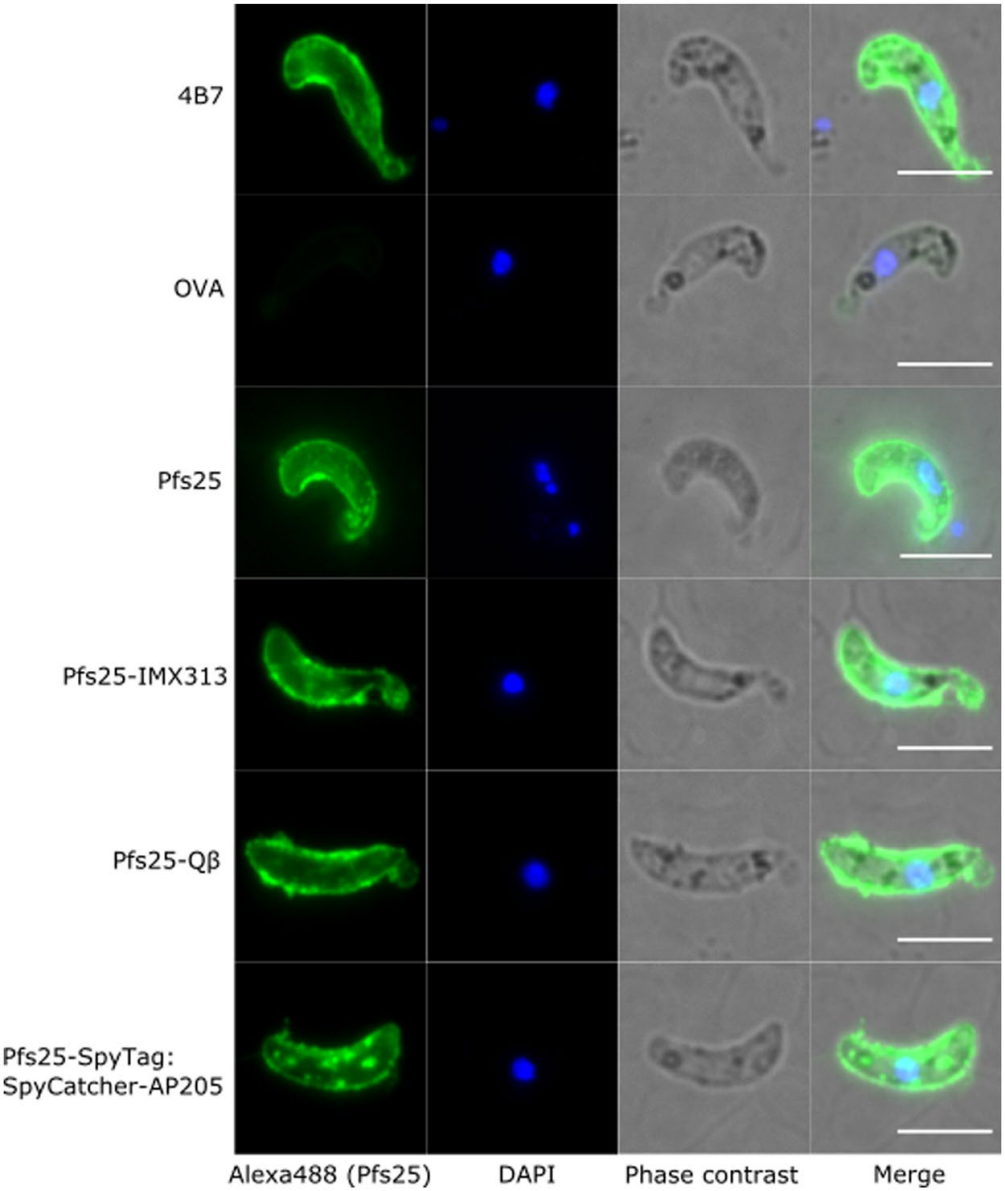
Binding of anti-Pfs25 IgG to native antigen. Ookinetes of *P*. *berghei* transgenic for Pfs25 (Pfs25DR3) were stained with 4B7 (positive control) or pooled post boost sera from mice immunised with one of the nanoparticle vaccines, monomeric Pfs25 or ovalbumin (OVA) (negative control). Antibody binding was detected by Alexa Fluor® 488-conjugated goat anti-mouse IgG (green) and the DNA was stained with DAPI (blue). Phase contrast as well as merged view are also shown. White bars correspond to 5 μm.

Expression of Pfs25 occurs on the ookinete surface in the midgut of the mosquito vector^8^. Using *P. berghei* parasites transgenically expressing *P. falciparum* Pfs25 (Pfs25DR3), we assessed the ability of IgG induced by vaccination to recognise native antigen on the ookinete surface (Fig. 3). Pooled sera from each group recognised Pfs25 on the surface of the parasite in an identical pattern to the 4B7 positive control. Only weak staining was observed with the sera from mice vaccinated with monomeric Pfs25, likely due to the significantly lower immunogenicity of this vaccine, so this immunofluorescence assay was performed at a lower serum dilution than the nanoparticle immunised sera. Serum from ovalbumin-immunised mice was used as a negative control.

### Discordance between titer and functional efficacy of vaccine-induced antibody responses

**Figure 4:**
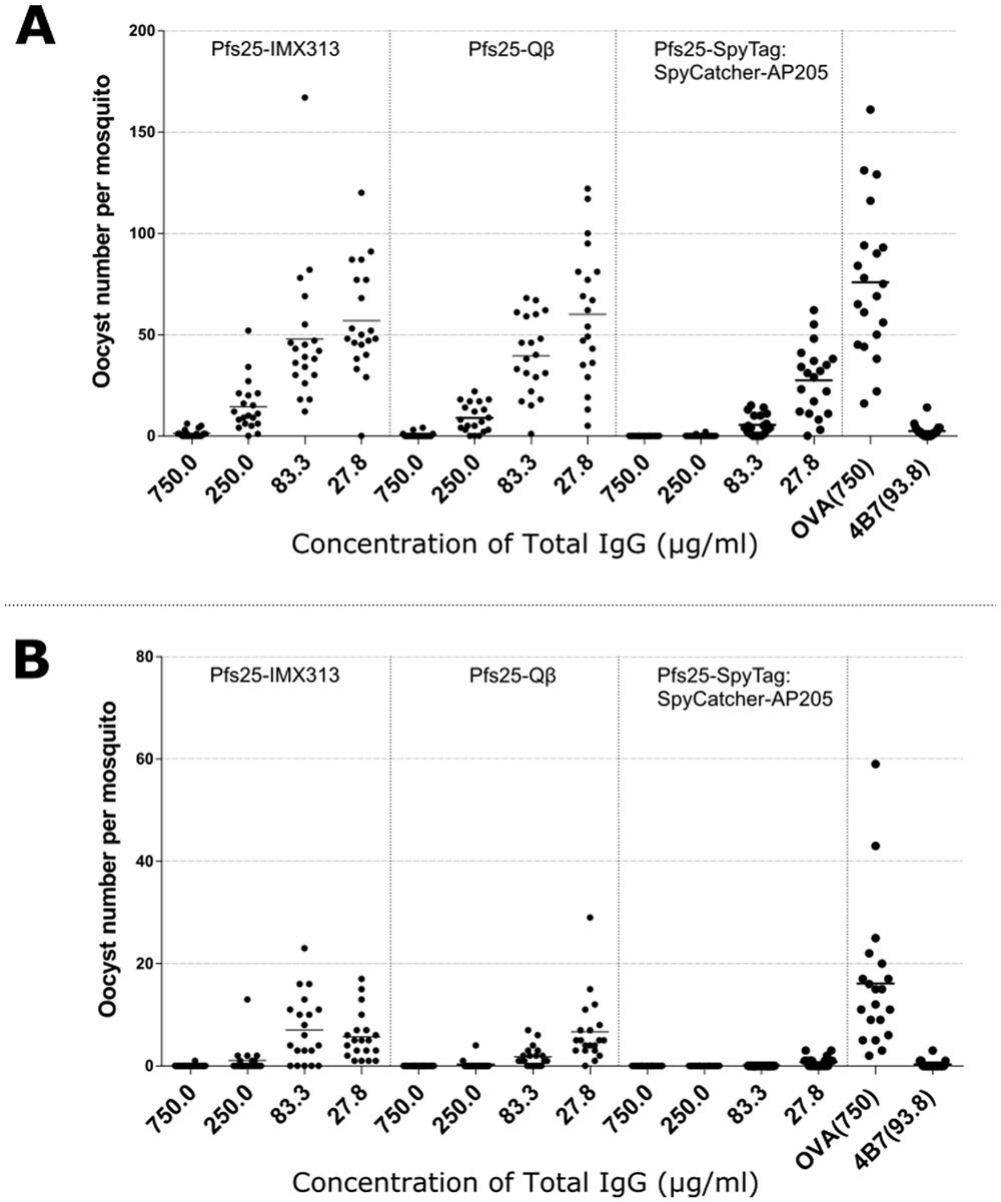
Transmission blocking efficacy of anti-Pfs25 IgG induced by nanoparticle vaccines. Total IgG was purified from the pooled serum of each group (3 weeks post boost). The purified IgG was mixed with *P. falciparum* NF54 cultured gametocytes and fed to *A. stephensi* mosquitoes (n = 20 per test group) in two independent SMFA experiments. (**A**,**B**) IgG from ovalbumin-immunized mice was used as a negative control; the transmission blocking anti-Pfs25 mAb 4B7 was used as a positive control. Midguts were dissected 7 days post-feeding. Data points represent the number of oocysts in individual mosquitoes and the lines show the arithmetic mean (**A**,**B**). x-axis values are μg/ml total IgG in the assay.

The functional efficacy of the immune response generated by the different vaccines was determined by standard membrane feeding assay (SMFA). This assay involves feeding live mosquitoes with a mixture of *P. falciparum* malaria-infected blood and purified IgG from each group, with dissection in order to determine the number of oocysts which develop^36^. Functional efficacy is determined by a reduction in the number of oocysts compared to a negative control group lacking protective antibody, this is termed the transmission reducing activity (TRA). In the first feeding assay (Feed #1), all purified IgG from the pooled serum of each group was tested at a single concentration of 750 μg/ml (Table 2 and Fig. S2). This assay demonstrated that the anti-Pfs25 IgG induced by vaccination with each of the nanoparticles had functional transmission-reducing activity (TRA) (Table 2 and Fig. S2). Unsurprisingly the TRA of the anti-Pfs25 IgG induced by monomeric Pfs25 was significantly lower in this experiment (Table 2 and Fig. S2, p = 0.184). Therefore, in the following two feeds (Feed #2 and Feed #3), purified IgGs of the Pfs25-IMX313, Pfs25-Qβ and Pfs25-SpyTag:SpyCatcher-AP205 groups were evaluated at three-fold dilutions starting at 750 μg/ml (750, 250, 83.3 & 27.7 μg/ml), to dissect whether there was a qualitative difference in transmission reducing activity between the anti-Pfs25 IgG induced by the three nanoparticle vaccines (Table 2 and Fig. 4).

**Table 2:** ﻿Transmission﻿ reducing activity of IgG from immunised mice﻿.

Sample	Conc^a^	Mean Ooc^b^	% inhibition^c^	p value^d^
**Feed #1**				
4B7	93.75	0.5	98.4 (95, 99.5)	<0.001
OVA	750	26.2	N/A	N/A
Pfs25	750	12.7	51.6 (−35.3, 83.7)	0.184
Pfs25-IMX313	750	0	100 (99.3, 100)	<0.001
Pfs25-Qβ	750	0.1	99.8 (98.7, 100)	<0.001
Pfs25-SpyTag:SpyCatcher-AP205	750	0	100 (99.4, 100)	<0.001
**Feed #2**				
4B7	93.75	2.35	97.9 (93.9, 99.3)	<0.001
OVA	750	75.85	N/A	N/A
Pfs25-IMX313	750	1.2	98.4 (95.2, 99.5)	<0.001
	250	14.35	81.1 (45.3, 93.6)	0.004
	83.3	47.75	37 (−74.8, 76.2)	0.406
	27.8	56.8	25.1 (−123.3, 76.1)	0.598
Pfs25-Qβ	750	0.45	99.4 (98, 99.9)	<0.001
	250	8.85	88.3 (66.1, 96.1)	<0.001
	83.3	39.6	47.8 (−58, 82.1)	0.249
	27.8	60.05	20.8 (−121.5, 72.3)	0.664
Pfs25-SpyTag: SpyCatcher-AP205	750	0	100 (99.7, 100)	<0.001
	250	0.15	99.8 (99.3,100)	<0.001
	83.3	5.4	92.9 (79.4, 97.6)	<0.001
	27.8	27.45	63.8 (−1, 87.4)	0.051
**Feed #3**				
4B7	93.75	0.3	98.4 (95.2, 99.6)	<0.001
OVA	750	16.1	N/A	N/A
Pfs25-IMX313	750	0.05	99.7 (98.8, 100)	<0.001
	250	1.1	93.2 (77.3, 98.7)	<0.001
	83.3	7.05	56.2 (−33.1, 85.5)	0.152
	27.8	5.75	64.3 (−11.5, 88.8)	0.07
Pfs25-Qβ	750	0	100 (98.8, 100)	<0.001
	250	0.25	98.4 (94.6, 100)	<0.001
	83.3	1.8	88.8 (63.7, 96.5)	<0.001
	27.8	6.65	58.7 (−30.2, 88.6)	0.131
Pfs25-SpyTag: SpyCatcher-AP205	750	0	100 (99, 100)	<0.001
	250	0	100 (99.3, 100)	<0.001
	83.3	0	100 (99, 100)	<0.001
	27.8	0.75	95.3 (83.1, 100)	<0.001

^a^IgG concentration (μg/ml) in feeder.

^b^Arithmetic mean of oocyst intensity from 20 mosquitoes.

^c^Percent inhibition of mean oocyst intensity and the 95% confidence interval (95% CI).

^d^Two-sided p values testing whether % inhibition is significantly different from zero.

Combined analysis of Feeds #2 and #3 demonstrated that, Pfs25-SpyTag:SpyCatcher-AP205 had statistically lower oocysts (i.e., higher TRA, Holm adjusted p-values < 0.042) than either Pfs25-IMX313 or Pfs25-Qβ except at the 750 μg/ml dilution (p = 0.042 to Pfs25-IMX313, but p = 0.213 to Pfs25-Qβ) when tested at the same total IgG concentrations. At 83.3 and 250 μg/ml, Pfs25-Qβ showed significantly lower oocysts (p = 0.030 and 0.020, respectively) than Pfs25-IMX313 (Table 2 and Fig. 4).

**Figure 5:**
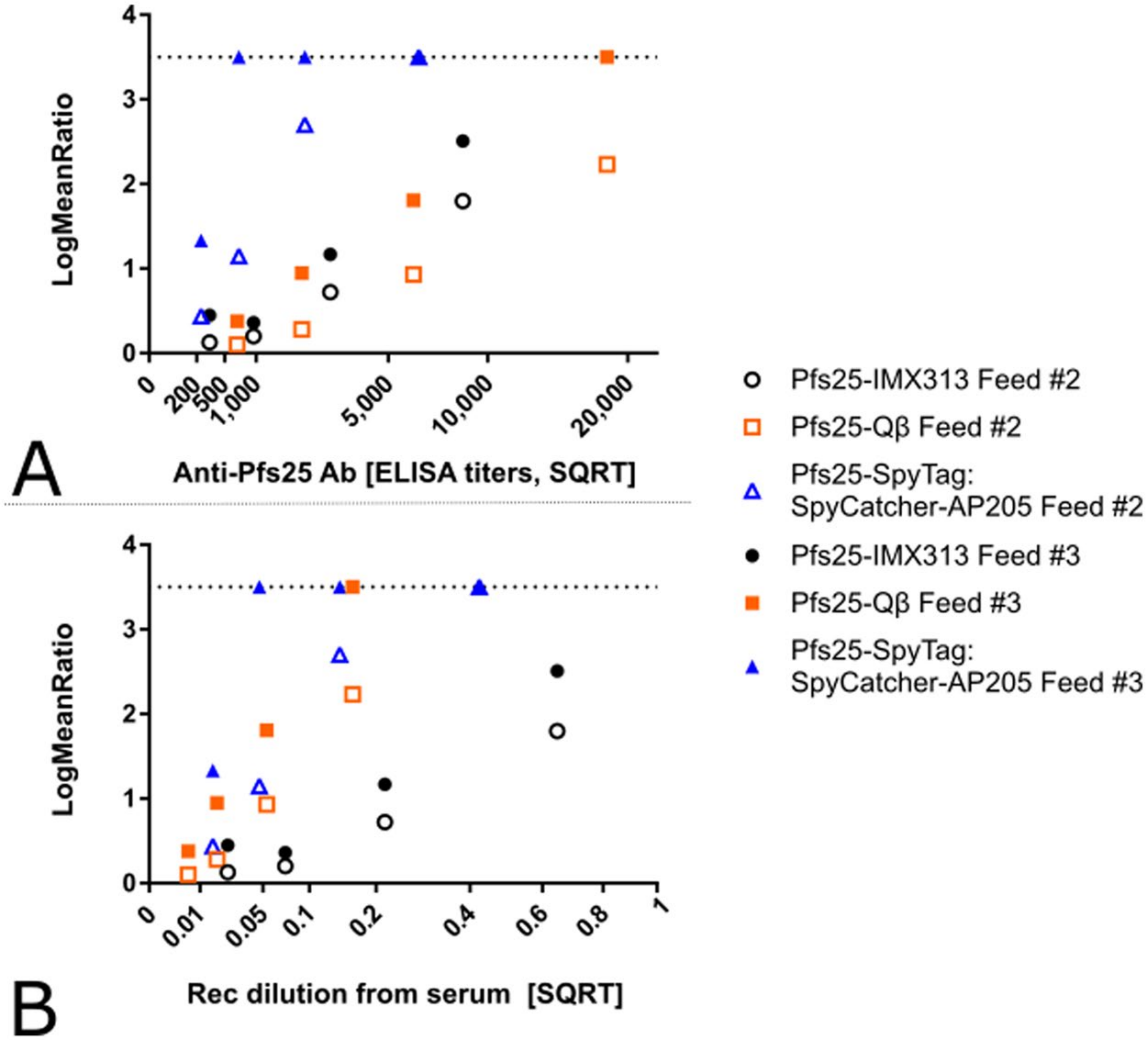
Comparison of the quality of the anti-Pfs25 IgG induced by nanoparticle vaccines. (**A**) The square root of anti-Pfs25 specific IgG level in the feeder is shown on the x-axis. The ratio of mean oocyst counts in control and test samples are plotted on a log scale along (log-mean ratio, LMR) the y-axis. (**B**) The reciprocal of the dilution factor from the original serum pool is plotted on the x-axis. LMR is plotted on the y-axis. When a test sample showed 100% inhibition in the SMFA, the LMR was assigned as 3.5 (dotted line) in the figures.

Further dissection of the functional efficacy of the anti-Pfs25 IgG induced by each platform is facilitated by determining the relationship between anti-Pfs25 antibody titers in the purified IgG used and the SMFA results. In a multiple linear regression analysis, anti-Pfs25 and vaccine groups were used as explanatory variables to determine the “transmission blocking efficacy per anti-Pfs25 antibody unit” (Fig. 5A). The overall fit to the linear regression model was R^2^ = 0.72, and both anti-Pfs25 AU (p < 0.0001) and vaccine groups (p = 0.0018) showed significant effects. Among the three vaccines, the Pfs25-SpyTag:SpyCatcher-AP205 group showed significantly higher activity after adjusting AU (p = 0.0057 to Pfs25-IMX313, and p = 0.0016 to Pfs25-Qβ), but there was no significant difference between Pfs25-IMX313 and Pfs25-Qβ (p = 0.4754).

We further determined “transmission reducing efficacy per volume (ml) of serum” using the dilution factor of the serum pool in a feeder (more specifically, reciprocal of dilution) as one of the explanatory variables, instead of anti-Pfs25 AU (Fig. 5B). In other words, we tried to evaluate how efficacious is each response in the undiluted mouse serum from each group (to assess the combined consequences of antibody quantity and quality). The overall fit to the linear regression model was R^2^ = 0.70, and both dilution factor (p < 0.0001) and vaccine groups (p = 0.0016) showed significant effects. When three groups were compared, Pfs25-Qβ and Pfs25-SpyTag:SpyCatcher-AP205 were able to induce a similarly efficacious response (p = 0.4512). Both of these vaccines were significantly better than Pfs25-IMX313 (Fig. 5B, p = 0.0094 and 0.0019, respectively). The similarity between Pfs25-Qβ and Pfs25-SpyTag:SpyCatcher-AP205 is due to the fact that, while Pfs25-Qβ induced a higher titer (Fig. 2A), the response was less efficacious per antibody unit (Fig. 5A).

## Discussion

In this study we have successfully generated and characterised Pfs25-Qβ VLPs (Fig. 1A). We further established that chemically crosslinking Pfs25 to VLPs of bacteriophage Qβ coat protein increases its immunogenicity significantly (Fig. 2A), as well as generating significantly higher avidity antibodies (Fig. 2B). This is consistent with previous reports that crosslinking peptides/antigens to Qβ VLPs improves immunogenicity^28–30^ as well as with reports that the immunogenicity of Pfs25 is improved by conjugation to VLPs or protein nanoparticles^4, 6, 9, 25^.

Furthermore, we successfully replicated previous reports of immunogenicity enhancement using IMX313 and SpyCatcher-AP205 platforms^4, 6, 25^ (Fig. 2). Unsurprisingly monomeric Pfs25 was significantly less immunogenic than any of the protein-nanoparticle platforms studied (Fig. 2). Side-by-side comparison of the three platforms displaying Pfs25 revealed some differences in both the magnitude (Fig. 2A) of the immune response as well as the efficacy of that response (Figs 4 and 5). Qβ not only increased immunogenicity compared to monomeric Pfs25 but ELISA titers were approximately a log higher than the titers at the corresponding time points from both other platforms (Fig. 2A). Similarly Qβ elicited a humoral response with significantly higher antibody avidity than Pfs25-IMX313 (Fig. 2B).

Functional efficacy measured by SMFA is the true readout of this study; SMFA is the gold standard for testing TBV candidates^36^. In SMFA, it is well appreciated that the error of assay is larger, especially when a weaker sample is tested in a single assay. It is true not only in our facility, but also in other laboratories^37^. Two main readouts are commonly given from SMFA: % inhibition in mean oocyst count (TRA) and % inhibition in prevalence (transmission blocking activity or TBA). However these readouts cannot be compared unless the mean number of oocysts in the controls are similar, and TBA is the function of TRA and mean control oocysts^37^. Therefore, we reported 95% CI for each % inhibition estimate (Table 2) and analysed data by combining multiple feeds at multiple concentrations (Fig. 4). Using this assay and combining the outcome with the antibody titers measured by ELISA we are able to determine the per-AU efficacy induced by each platform (Fig. 5A). We reproducibly demonstrated the antibodies induced by SpyCatcher-AP205 were able to block transmission much more strongly than those elicited by either IMX313 or Qβ at the same anti-Pfs25 AU. Indeed these other two platforms nearly overlapped in their per-AU efficacy (Fig. 5A). This result indicates that the antibodies to Pfs25 in the mice immunised with Pfs25-SpyTag:SpyCatcher-AP205 must be restricted to a more functionally relevant subset of epitopes, i.e. are more likely to block the Pfs25 function that is required for establishing infection in the mosquito midgut.

The cause of this effect is likely twofold: first chemical modification of Pfs25 to allow conjugation to Qβ at least partially unfolds the protein. The monoclonal antibody 4B7 has been demonstrated to bind to the reduced form of recombinant Pfs25 more strongly than to non-reduced Pfs25^4^, implying that it binds to a reducible unfolded epitope. As we demonstrated by Western blot, (Fig. S1) 4B7 binds more strongly to the thiolated-Pfs25 than to unthiolated-Pfs25, strongly suggesting protein unfolding. This unfolded protein conjugated to the Qβ-VLP will likely stimulate generation of antibodies to these partially unfolded epitopes distracting the immune response from relevant functional ones. Secondly display on AP205 is ordered: each Pfs25 molecule is anchored in the same C-terminal manner similar to its predicted display on the native parasite surface. Native Pfs25 is anchored by a C-terminal GPI-anchor^8^ and this is mimicked by display on SpyCatcher-AP205. Display on Qβ is disordered, essentially taking place between functionalised amine residues, meaning there are up to 25 possible conjugation sites on Pfs25^8^, though it is possible that not all are surface exposed. As a result Pfs25 is potentially displayed on the surface of Qβ in one of up to 25 orientations. Though it is difficult to determine the exact antigen density on the surface of Qβ, the fact that some monomeric Qβ coat protein (~14 kDa, Fig. 1C and Fig. S1) is detectable by reducing Coomassie-stained SDS-PAGE indicates that the coupling density is less than 100%. Conversely 100% coupling, i.e. each coat protein molecule is linked to a Pfs25-SpyTag molecule, of Pfs25-SpyTag to SpyCatcher-AP205 is readily evident (Fig. 1B). The combination of these factors is likely to explain the differences in functional efficacy observed between Qβ and SpyCatcher-AP205.

The differences between SpyCatcher-AP205 and IMX313 are probably explained in a similar fashion. While Pfs25 is fused at its C-terminus to IMX313 the fact that there are only ~7 molecules of Pfs25 per nanoparticle means that functional epitopes are arrayed differently to those on SpyCatcher-AP205. Extrapolating from the structure of C4bp we expect Pfs25-IMX313 to form a heptagonal flat disc^4, 38, 39^, whereby Pfs25 is displayed at the vertices of the disc exposing functionally non-relevant epitopes on each molecule’s side.

When functional efficacy of each platform is related to the hypothetical serum dilution used in the SMFA, we again establish that SpyCatcher-AP205 induces the most functionally efficacious response, though the differences between SpyCatcher-AP205 and Qβ are not as striking in this analysis. This reinforces the contention that antibodies induced by SpyCatcher-AP205 are more functionally relevant for transmission blocking. Even though per-ml of serum there are fewer anti-Pfs25 antibodies; since the proportion of total anti-Pfs25 response that is functionally relevant is much higher, less serum is required to effectively block transmission.

Differences in the IgG subclass elicited by each platform are worth considering as a potential source of the differences in functional efficacy that we observe in this study. However, in contrast to anti-Pfs230 antibodies, there is no report showing that anti-Pfs25 antibodies require complement to show functional efficacy in SMFA^40–42^. Indeed in this and previous studies to assess Pfs25 vaccine efficacy, we have performed the SMFA using heat-inactivated serum^4, 6^ thereby precluding a role for complement. Therefore, we deem it unlikely that IgG subclass profile has a significant impact on functional efficacy measured in the SMFA in the case of Pfs25 vaccines.

To date four Pfs25 candidate vaccines have progressed to Phase Ia clinical trials (Pfs25-EPA conjugate, Pfs25 VLP-FhCMB (Fraunhofer USA Center for Molecular Biotechnology), Chimpanzee Adenovirus 63 (ChAd63) Pfs25-IMX313/Modified vaccinia Ankara (MVA) Pfs25-IMX313, and Pfs25-Pfs25 conjugate; clinicaltrials.gov search term “Pfs25”) and there are a number of others in pre-clinical development^4, 6, 9, 25^. We have previously demonstrated an improvement in the functional quality of the antibody response elicited by vaccination with Pfs25-IMX313 over monomeric Pfs25^4^. This study confirms that there is significant value in analysing not only the immunogenicity and functional efficacy of a candidate vaccine but also the relationship between the two, and this should inform the design of future clinical trials.

Of the display technologies used in this study, to date only a Qβ VLP has been tested in clinical trials (NCT00369616 & NCT01280968), as an anti-nicotine immunotherapy. These studies were unable to detect a statistically significant difference in long-term smoking cessation between participants receiving vaccine and those receiving placebo^43^. Nicotine is a small molecule alkaloid and as such a conjugation to Qβ is unlikely to affect its structure, in contrast to a protein conjugate which has secondary and tertiary structure which can be disrupted by chemical conjugation. No nicotine addiction vaccine trial has to date demonstrated an improvement in smoking cessation, Pfs25 vaccines have however demonstrated functional efficacy^44, 45^. The clinical results of the Qβ-nicotine VLP unfortunately therefore do not particularly inform its relevance to the field of protein-subunit vaccines: however the results of our study likely eliminate its selection as a candidate platform for a Pfs25 vaccine.

Of the other platforms tested in this study, IMX313 has completed one first-in-human phase 1 trial to evaluate the safety and immunogenicity of a candidate tuberculosis vaccine expressed via viral vectors rather than as a protein^46^. Pfs25-IMX313 is currently undergoing a phase Ia clinical trial in a ChAd63-MVA viral vectored formulation (NCT02532049). We have previously demonstrated that in this formulation IMX313 does not significantly improve the quality of the vaccine-induced immune response but does increase its magnitude^4^; results of this trial are expected in early 2018. Conversely Pfs25-IMX313 does improve the quality of the vaccine induced immune response when delivered as a protein-in-adjuvant in pre-clinical studies^4^. Neither AP205 nor SpyCatcher have any available clinical data.

Taken together these results demonstrate that improving the number of antibodies to a vaccine antigen should not be the only consideration when selecting an antigen display technology such as a VLP or nanoparticle; the quality of the response induced by vaccination is of major importance. The importance of antibody *quality* has been shown previously for vaccines for other infectious diseases^47–50^, as well as during natural responses to infection^51, 52^. Platform technologies which improve antibody titer must therefore be carefully compared and considered in order to determine which will improve the candidate vaccine. If simply improving antibody titer to any part of the antigen is sufficient then Qβ may be the best platform. However if a focused response is required to the antigen in a specific orientation, then SpyCatcher-AP205 is a superior platform. The biochemistry underpinning SpyCatcher-SpyTag allows versatile antigen coupling orientation by changing the position of SpyTag, which can be placed at either terminus of a candidate antigen or even within surface exposed flexible loops^53^. This would allow orientation on the VLP surface to be specifically chosen to display the most functional epitopes.

Future work to determine whether this property is specific to SpyCatcher conjugation on the surface of bacteriophage VLPs or is a generalizable property of SpyCatcher-VLP fusions will be of value to the field of vaccinology. If it is indeed a generalizable property, it will be useful to demonstrate efficacy on as many VLPs as possible in order to create a diverse library of potential scaffold technologies. A highly effective malaria vaccine is likely to incorporate elements targeting multiple stages of the parasite life-cycle, and transmission blocking vaccines will ultimately be a crucial component. A clear understanding of the quantity and quality of the immune response required for a TBV to be effective will be of great benefit to the field. Modular VLP technologies such as SpyCatcher-AP205 are a useful tool to help dissect that response and to bring an effective TBV closer to fruition.

## Experimental Procedures

### Expression and purification of proteins

Qβ VLPs were grown in JM109 *E. coli* cells containing the pQβ10 plasmid described previously^54^. A starter culture of 50 ml was grown overnight at 37 °C with shaking in lysogeny broth (LB) supplemented with 50 μg/ml carbenicillin. This culture was then inoculated into 450 ml of complete M9 media (11.28 g/L M9 minimal salts, 10 g/L acid-hydrolysed casein, 2 mM MgSO_4_, 1 g/L glucose, 50 μg/ml carbenicillin) and incubated at 37 °C with shaking for a further 24 h. Cells were pelleted at 6000 × *g* for 15 min at 4 °C and frozen at −20 °C overnight to aid lysis. The cell pellet was then resuspended in 20 ml lysis buffer (20 mM Na_2_HPO_4_, 0.1% triton-X 100, 5 mM EDTA, 2 mg/ml lysozyme, pH 7.5) and incubated at room temperature for 20 min before addition of benzonase at a concentration 100 U/g of cell pellet with a further incubation at room temperature for 20 min. This was then subjected to 5 cycles of freeze thaw in a dry ice/methanol bath. The cell debris were pelleted by centrifugation at 15,500 × *g* for 30 min at 4 °C and the resulting supernatant was filtered through 0.85, 0.45 and 0.22 μm filters in succession. This was mixed with an equal volume of 100% (NH_4_)_2_SO_4_ and incubated on ice for 1 h to precipitate the VLPs. Precipitated VLPs were pelleted by centrifugation at 15,500 × *g* for 90 min at 4 °C. The supernatant was removed and precipitated protein was resuspended in 50 ml of Qβ buffer (10 mM Tris-HCl, 5 mM EDTA, 150 mM NaCl, pH 8.0). Five ml fractions of the resuspended VLPs were run on a S500 HR size exclusion column (GE healthcare, UK) equilibrated in Qβ buffer and the fractions corresponding to the predicted size of VLPs were collected and pooled from subsequent runs. The pooled VLP-containing fractions were applied to a CHT type II 80 μm hydroxyapatite (Bio-Rad, UK) column to remove contaminating lipopolysaccharide. The column was equilibrated in PBS and the pooled S500 HR elution fractions were diluted in PBS until the conductivity was below 12 mS/cm and applied to the column at a flow rate of 1 ml/min. The column was washed with 10 column volumes of 20 mM Tris pH 7.2 and the VLPs were eluted with 500 mM Na_2_HPO_4_, 1 M NaCl, pH 7.2. The eluate was diluted in PBS until the conductivity was below 12 mS/cm and reapplied to the hydroxyapatite column after it had been cleaned with 1 M NaOH for 2 h. This was repeated as necessary until the endotoxin content of the elution fraction was below 1 EU per μg.

Pfs25, Pfs25-IMX313-CTag, Pfs25-SpyTag-CTag (GenBank accession number KU302811) and SpyCatcher-AP205 (GenBank accession number KU302810) were purified as described previously^4, 6^. Each form of Pfs25 expressed and used in this study comprises the sequence from Alanine-22 to Threonine-193 with four potential N-linked glycosylation sites (112, 165, 187 and 202) mutated (as described previously)^4, 55^. Briefly: Pfs25-His and Pfs25-IMX313-CTag were expressed in *Pichia pastoris* under the control of the methanol inducible AOX1 promoter and alpha-mating factor secretion signal sequence allowing inducible expression and secretion into the culture media. Following expression both proteins were purified from the culture supernatant using either a 5 ml HisTrap HP nickel chelate affinity chromatography column (GE Healthcare) for Pfs25-His or a 5 ml CaptureSelect™ C-tag affinity matrix column for Pfs25-IMX313-CTag. Pfs25-SpyTag-CTag was expressed in HEK293E cells cultured to 2 million cells/mL and transfected with 1 μg plasmid complexed to polyethylenimine per million cells. Supernatant was harvested after 72 h and Pfs25-SpyTag-CTag was purified using a 5 ml CaptureSelect™ C-tag affinity matrix column. Pfs25-His, Pfs25-IMX313 and Pfs25-SpyTag-CTag were further polished by size exclusion chromatography using a HiLoad 16/600 Superdex pg column (GE Healthcare). SpyCatcher-AP205 was expressed intracellularly in C41 *E. coli* using an IPTG inducible promoter. VLPs were purified from the bacterial cell lysates using a Ni-NTA agarose resin (Qiagen) and polished by dialysis in a 300 kDa MWCO membrane (SpectrumLabs). Ni-NTA conditions were adjusted such that monomeric SpyCatcher-AP205 was washed off the column at a low imidazole concentrations (200 mM) and only intact VLPs were retained on the column until elution (2 M imidazole) as previously described^6^.

Western blots were performed using standard methods^4^. Briefly, after polyacrylamide gel electrophoresis and transfer to blotting membrane, blots were placed in an iBind (Thermo Fisher scientific, UK) apparatus according to the manufacturer’s instructions. Primary antibody (polyclonal or monoclonal) and secondary antibody (alkaline phosphatase conjugated donkey-anti-mouse IgG secondary antibodies [Jackson Immuno Research, USA]) probes were diluted to appropriate concentrations in iBind buffer. After washing briefly in deionized water, blots were incubated with 1 SIGMAFAST™ BCIP® /NBT tablet (Sigma-Aldrich, UK) dissolved in 10 ml dH_2_O until the desired level of staining was achieved.

### Antigen-VLP conjugation

Qβ and Pfs25-His prepared as above were buffer exchanged into PBS by dialysis and concentrated to 2 mg/ml, and 3 mg/ml respectively using a 100 kDa MWCO membrane centrifugal spin filter (Millipore). Pfs25-His was incubated with a 5-fold molar excess of N-succinimidyl S-acetylthioacetate (SATA) (Pierce, UK) at room temperature for 30 min. The latent sulfhydryls on the SATA-modified protein were de-protected with 10 mg/ml Hydroxylamine-HCl (Pierce, UK) for 3 h at room temperature. During this time Qβ VLPs were modified with a 10-fold molar excess of Succinimidyl 6-((β-maleimidopropionamido)hexanoate) (SMPH) (Pierce, UK) and incubated at room temperature for 2 h. Residual SMPH and SATA were removed from both by buffer exchange in a centrifugal spin-filter (Millipore, UK) of the relevant MWCO. The resulting thiolated-Pfs25 was mixed with derivatised-Qβ at a molar ratio of 5:1 and incubated at room temperature for 2 h. Remaining unconjugated antigen was removed by dialysis against TBS (20 mM Tris, 150 mM NaCl, pH 7.4) three times for 3 h using a 100 kDa MWCO membrane.

Pfs25-SpyTag-CTag and SpyCatcher-AP205 were conjugated as previously described^6^. Briefly, SpyCatcher-VLPs (30 μM) were incubated with 1.5 fold molar excess of Pfs25-SpyTag for 3 h at RT with addition of 10 × reaction buffer (40 mM Na_2_HPO_4_, 200 mM sodium citrate, pH 6.2). The reaction was then dialyzed with a 300 kDa cut-off membrane three times against 1,000-fold excess 50 mM glycine, 25 mM sodium citrate, 0.1% (v/v) Tween 20, pH 8.0 to remove unreacted Pfs25-SpyTag.

### VLP characterisation by size exclusion chromatography

Antigen-conjugated nanoparticles were applied to a previously equilibrated Superdex 200 Increase 10/300 GL (GE Healthcare, UK) on an AKTA pure fast protein liquid chromatography (FPLC) system (GE Healthcare). The mobile-phase column buffer was 20 mM Tris-HCl, 150 M NaCl, pH 7.4 and the applied flow-rate was 0.75 mL/min, all at 21 °C. For calibration, a high molecular weight gel filtration standard (GE Healthcare) was used.

### Transmission electron microscopy (TEM)

10 μL of VLPs (0.2 mg/mL) were applied to freshly glow-discharged carbon 200 mesh copper grids for 2 min, blotted with filter paper, and stained with 2% uranyl acetate for 10 s, then blotted and air dried. Grids were imaged in a FEI Tecnai T12 transmission electron microscope at 120 kV using a Gatan US1000 CCD camera.

### Vaccinations

All animal experiments and procedures were performed according to the UK Animals (Scientific Procedures) Act Project Licence (PPL 30/2414 and 30/2889) and approved by the Oxford University Local Ethical Review Body. For all vaccinations, a minimum specification of <1 EU/µg was used as a quality control to prevent endotoxin related reactogenicity. Age-matched female BALB/c mice (Harlan, UK), housed in specific pathogen-free environments, were vaccinated with 25 μl of vaccine formulation in each leg intramuscularly (IM). Protein-in-adjuvant was formulated as follows: 85 μg Alhydrogel (Brenntag) per dose was mixed with TBS at room temperature for 15 min, antigen was then added and incubated for a further 45 min at room temperature. Dosages were calculated to achieve a final concentration of 1 μg of Pfs25 per dose. Total protein per dose for each group is detailed in Table 1. The animals were immunised on days 0 and 21, and blood samples were collected on days 20 (three weeks post prime) and 41 (three weeks post boost). The samples were allowed to clot at 4 °C overnight before centrifugation at 13,000 × *g* in a benchtop centrifuge and serum was removed for testing.

### Immunofluorescence Assay

Recognition of native parasite Pfs25 antigen by the vaccine-induced antiserum was confirmed by immunofluorescence on paraformaldehyde (PFA) fixed ookinetes as described previously^56^. Briefly, Pfs25DR3 transgenic *Plasmodium berghei* (provided by Dr Andrew Blagborough, Imperial College, London) ookinete culture smears were fixed for 10 min in 4% PFA/PBS followed by washing in PBS. Slides were blocked for 1 h in blocking buffer (3% BSA in PBS) followed by incubation in test antiserum (1:500 for nanoparticles or 1:100 for monomeric Pfs25) or positive control antibody 4B7 ^32^ (1:500) diluted in 3% BSA in PBS for 45 minutes. Alexa-Fluor 488-conjugated goat anti-mouse IgG (Thermo Fisher, UK) (1:800) diluted in 3% BSA in PBS was then added and incubated for a further 45 minutes. Slides were then mounted in Mowiol-DAPI (Sigma-Aldrich, UK), a cover slip was added and the mounting media was allowed to set overnight at 4 °C in a slide box. Unless otherwise stated, all of the above steps were followed by 3 washes for 5 min in PBS and incubations were performed at room temperature in a humid chamber. Slides were analysed by fluorescence microscopy on a DMI3000B microscope (Leica Microsystems, UK).

### Pfs25 standardised ELISA

The protocol used to assess anti-Pfs25 antibody titers in serum has been described previously^4^. Briefly, Nunc-Immuno maxisorp plates (Thermo Scientific, UK) were coated with 0.1 μg (100 μl of 1 μg/ml stock) *P. pastoris* expressed Pfs25-His per well in coating buffer (50 mM sodium bicarbonate, pH 9.6) and incubated overnight at 4 °C. Plates were washed with PBS + 0.1% Tween-20 (PBS/T) six times and blocked for 1 h with 5% skimmed milk in PBS/T at room temperature (RT). Test serum samples were diluted as required in PBS/T, before 100 μl of sample were added to triplicate wells and incubated for 2 h at RT. Plates were washed as before and 100 μl donkey anti-mouse total IgG conjugated to alkaline phosphatase (Sigma-Aldrich, UK) diluted 1:3000 in PBS/T was added to each well and incubated for 1 h at RT. Following a final wash in PBS/T, one p-nitrophenylphosphate (Sigma-Aldrich, UK) tablet was dissolved in 1x diethanolamine buffer (Thermo Scientific, UK) and 100 μl added to each well as a developing substrate. The optical density (OD) of each well was read at 405 nm using an ELx800 absorbance microplate reader (Biotek, UK). A serially diluted standard reference serum (as previously reported^4^) with a known antibody titer was used to determine the antibody titer of individual samples. The minimal detection limit (MDL) of the ELISA was 100 AU/ml. Samples which fell below this value were defined as having 1 AU/ml in order to display them on a log scale graph.

### Antibody Avidity ELISA

Antibody avidity (three weeks post boost) was assessed using a sodium thiocyanate (NaSCN)-displacement ELISA. Nunc-Immuno maxisorp plates were coated with recombinant Pfs25-His protein over-night, blocked and then washed with PBS/T as described for standardised ELISA. All individual serum samples were diluted so that each sample contained the same level of Pfs25 AU (in this study 100 AU). Samples were added in duplicate and following incubation and washing, an ascending concentration of the NaSCN (0–7 M) was added to the wells. The plates were incubated at RT for 15 min followed by washing and further development as in the standardised ELISA.

### IgG Purification

To perform standard membrane feeding assays (SMFAs), mouse sera were pooled and the IgG purified, as previously described^10^. Mouse sera from the final bleeds post-immunisation were pooled within each test and control group. Equal volumes of serum from all mice in a group were pooled irrespective of individual antibody titer. Total IgG was purified using Protein G columns (Pierce, USA) as described previously^4^ and buffer exchanged to 1x PBS.

### Standard Membrane Feeding Assay

The ability of vaccine-induced antibodies to block the development of *P. falciparum* strain NF54 was evaluated by SMFA as previously described^13^. The percentage of mature Stage V gametocytes was adjusted to 0.15% ± 0.05% and the male-female ratio is stable (almost always 1 male: 2–3 female). Gametocyte cultures were mixed with purified IgG at the concentrations (diluted in PBS) shown in the figures and then fed to 4–6 day old starved female *Anopheles stephensi* (SDA 500) via a parafilm® membrane. The mosquitoes were maintained at 26 °C and 80% relative humidity. After 7 days, midguts from twenty mosquitoes with eggs per group were dissected, oocysts counted and the number of infected mosquitoes recorded. Percent reduction in infection intensity was calculated relative to the respective control IgG tested in the same assay.

### Statistical Analysis

Comparison of quantitative data from multiple groups (e.g., antibody titers) was performed using a Kruskal-Wallis test. If significant, a Dunn’s multiple comparison post-test was performed. Avidity ELISA data of each sample were fitted to a sigmoidal dose-response (variable slope) curve and the EC50 was defined as the concentration of NaSCN required to reduce the OD by 50% between the upper and lower limits.

For SMFA data, 20 mosquitoes per group were analysed and the number of mosquitoes is reflected in the 95% confidence intervals (CIs) of % inhibition. The best estimate and 95% CIs of % inhibition in oocyst density from a single or multiple feeding experiments for each test antibody at each concentration were calculated using a negative binomial model with zero inflation model, as described previously^13^.Oocyst densities of two groups were compared using the same model, and Holm corrected p-values were calculated for multiple comparisons.

A linear regression model was used to evaluate difference in functional activity among vaccine groups after adjusting other factors^4^. The log10 transformed ratio of the mean oocyst count in control and test samples was the dependent variable, and the square root of anti-Pfs25 AU and vaccine group were independent variables in the model. When a test sample showed 100% inhibition, the data point was excluded from the analysis, as it was outside of linear range (i.e., log-transformed values became infinity). If the vaccine group effect was significant, Tukey’s honest significant difference tests were performed to compare different groups. In another linear regression analysis, reciprocal of dilution (in a square root scale) from the original pooled serum to the tested IgG in a feeder was utilised as one of the independent variables, instead of anti-Pfs25 AU. Since the ratio of anti-Pfs25-specific IgG to entire IgG in a sample is considered to be stable before (i.e., in the original pooled serum) and after (i.e., in the purified IgG) protein G affinity purification, the anti-Pfs25 AU in the original pooled serum and in the purified IgG were used to calculate the dilution factor of each test IgG at each test concentration.

Statistical tests were performed using Prism 6 (GraphPad Software Inc, USA), or JMP11 (SAS Institute Inc, USA). P-values < 0.05 were considered significant.

## Electronic supplementary material

Supplementary Figures
