# Supplementary material for: Nanoassembly routes stimulate conflicting antibody quantity and quality for transmission-blocking malaria vaccines
Source: Sci Rep. 2017 Jun 19;7:3811. doi: 10.1038/s41598-017-03798-3 (PMC5476561; doi:10.1038/s41598-017-03798-3)
Supplement: Supplementary file 1 — Supplementary Figures [file 41598_2017_3798_MOESM1_ESM.pdf]

Title: Nanoassembly routes stimulate conflicting antibody quantity and quality for transmission blocking malaria vaccines.

Supplementary Figures:

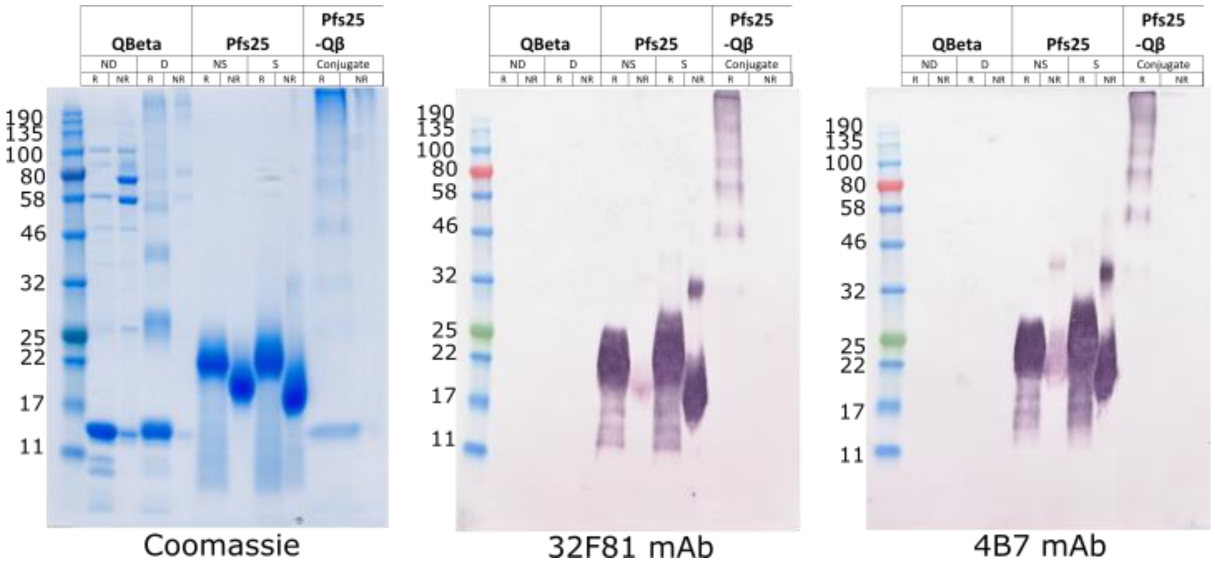

Figure S1. SDS-PAGE and western blot analysis of chemical modification and conjugation of Pfs25 and Qβ. Coomassie stained reducing (R) and non-reducing (NR) SDS-PAGE of pre- (ND) and post-derivatisation (D) Qβ coat protein, pre- (NS) and post-sulphydryl addition (S) Pfs25 and conjugated (conjugate) Pfs25-Qβ. Western blot analysis of the same samples was performed using both conformational, transmission blocking mAbs 4B7 and 32F81. Numbers on the left of each panel indicate size markers in kDa.

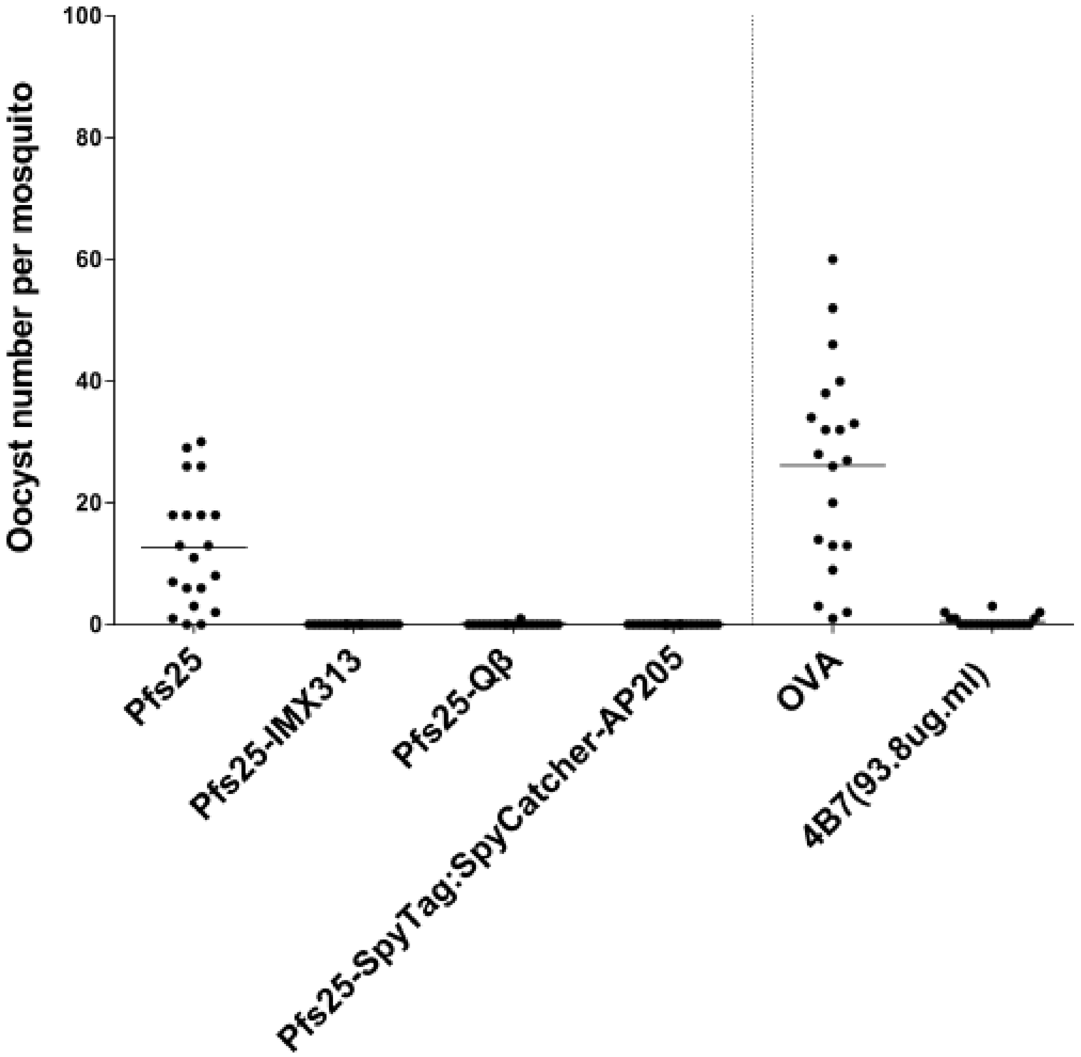

Figure S2. Transmission blocking efficacy of anti-Pfs25 IgG induced by nanoparticle vaccines. Total IgG was purified from the pooled serum of each group (3 weeks post boost). The purified IgG was mixed with *P. falciparum* NF54 cultured gametocytes and fed to *A. stephensi* mosquitoes (n = 20 per test group). IgG from ovalbumin immunized mice was used as a negative control, the transmission blocking anti-Pfs25 mAb 4B7 was used as a positive control. Midguts were dissected 7 days post-feeding. Data points represent the number of oocysts in individual mosquitoes and the lines show the arithmetic mean All samples were tested at 750 μg/ml total IgG unless otherwise indicated.
